# Key Performance Indicators of Secondary Health Care in Chronic Kidney Disease: Experience in Public and Private Services in the State of São Paulo, Brazil

**DOI:** 10.1155/2024/5401633

**Published:** 2024-10-26

**Authors:** Farid Samaan, Cristiane Akemi Vicente, Luiz Antônio Coutinho Pais, Gianna Mastroianni Kirsztajn, Ricardo Sesso

**Affiliations:** ^1^Planning and Evaluation Group, State Department of Health of São Paulo, São Paulo 01246-901, Brazil; ^2^Special Programs, Hapvida-NotreDame Intermédica Group, São Paulo 03164-140, Brazil; ^3^Research Division, Dante Pazzanese Cardiology Institute, São Paulo 04012-909, Brazil; ^4^Nephrology Division, Federal University of São Paulo, São Paulo 04023-062, Brazil

**Keywords:** chronic kidney disease, multidisciplinary care, quality indicators in health care, secondary health care, supplementary health, unified health system

## Abstract

**Introduction:** The objective of this study was to evaluate quality indicators of secondary health care in chronic kidney disease (CKD).

**Methods:** This retrospective longitudinal study was conducted in an outpatient medical nephrology clinic of the Brazilian Unified Health System (UHS) and a multidisciplinary outpatient clinic of a private health plan (PHP). The inclusion criteria were age ≥ 18 years, ≥ 3 medical appointments, and follow-up time ≥ 6 months.

**Results:** Compared to PHP patients (*n* = 183), UHS patients (*n* = 276) were older (63.4 vs. 59.7 years, *p*=0.04), had more arterial hypertension (AH) (91.7% vs. 84.7%, *p*=0.02) and dyslipidemia (58.3 vs. 38.3%, *p* < 0.01), and had a lower estimated baseline glomerular filtration rate (eGFR) (29.9 [21.5–42.0] vs. 39.1 [28.6–54.8] mL/min/1.73 m^2^, *p* < 0.01). Compared to PHP patients, UHS patients had a lower percentage of diabetics with glycated hemoglobin < 7.5% (46.1% vs. 61.2%, *p*=0.03), fewer people with potassium < 5.5 mEq/L (90.4% vs. 95.6%, *p*=0.04), and fewer referrals for hemodialysis with functioning arteriovenous fistula (AVF) (9.1% vs. 54.3%, *p* < 0.01). The percentages of people with hypertension and blood pressure < 140 × 90 mmHg were similar between the UHS and PHP groups (59.7% vs. 66.7%; *p*=0.17), as was the percentage of people with parathyroid hormone control (85.6% vs. 84.8%; *p*=0.83), dyslipidemia and LDL-cholesterol < 100 mg/dL (38.3% vs. 49.3%; *p*=0.13), phosphorus < 4.5 mg/dL (78.5% vs. 72.0%; *p*=0.16), and 25-OH-vitamin-D > 30 ng/mL (28.4% vs. 36.5%; *p*=0.11). The crude reduction in eGFR was greater in the UHS group than in PHP (2.3 [−0.1; 5.9] vs. 1.1 [−1.9; 4.6] mL/min/1.73 m^2^; *p* < 0.01). In the multivariate linear mixed-effects model, UHS patients also showed faster CKD progression over time than PHS ones (group effect, *p* < 0.01; time effect, *p* < 0.01; interaction, *p* < 0.01).

**Conclusions:** Quality of care for patients with CKD can be improved through both services, and multidisciplinary care may have a positive impact on the control of comorbidities, the progression of CKD, and the planning of the initiation of hemodialysis.

## 1. Introduction

Chronic kidney disease (CKD) is a global public health problem due to the exponential increase in its prevalence and the costs of treatment [1]. The nations in demographic and epidemiological transition face a special challenge in the care of patients with CKD, mainly due to the increased longevity of individuals and the increasing rates of arterial hypertension (AH) and diabetes mellitus (DM) in the population, which are the main risk factors for CKD [2, 3].

Among these nations, Brazil stands out as a Latin American upper–middle-income country in which the percentage of people over 60 years has increased from 9% to 15% in the last two decades [4]. In the same period, the incidence rates of AH, DM, and obesity increased from 21% to 25%, from 5% to 9%, and from 11% to 23%, respectively [5]. In fact, Brazil is one of the 10 nations with the highest increase in the prevalence of CKD undergoing dialysis treatments and the fourth in the absolute number of patients with this condition [6].

The line of care for CKD is well defined by international and Brazilian guidelines, that is, the roles of primary, secondary (or specialized outpatient), and tertiary care are known [7–9]. In addition, multidisciplinary care is widely recognized to be associated with better outcomes for patients with advanced CKD [2, 7]. Since CKD can be prevented or mitigated with adequate care, monitoring the quality of care is essential for all health services [10].

Several studies have shown gaps in the quality of care of CKD patients at the primary and tertiary levels [11–13]. In Brazil, fewer studies have been published on the quality of secondary care for CKD. The country has a dual healthcare system in which the Unified Health System (UHS) (public and with universal access) coexists with the supplementary one (private) [14]. Approximately 25% of the Brazilian population has a private health plan (PHP) [15]. There are differences in health determinants between people with and without private health insurance; however, the implication of this phenomenon has not been determined in the Brazilian population with CKD [16, 17]. Therefore, the objective of this study was to evaluate quality indicators of secondary health care in CKD within two prominent health settings in the state of São Paulo, Brazil, one public and the other private.

## 2. Materials and Methods

### 2.1. Design, Population, and Inclusion and Exclusion Criteria

This was a retrospective, longitudinal study based on consultation of the records of patients treated at two secondary care services for CKD in the state of São Paulo, Brazil. One of the services was a specialty facility of the UHS, which uses a traditional care model, that is, based only on medical appointments. The other was a PHP service, which offers multidisciplinary care, including medical consultation with a nephrologist, followed by consultation with a nurse and nutritionist. In Brazil, all citizens can use the UHS without restrictions. The PHP that took part in this study is primarily contracted by companies for their employees and, to a lesser extent, through direct and voluntary contracting by individuals or their families. At the UHS nephrology outpatient clinic, patients are referred by general practitioners or physicians from other specialties according to the individual judgment of these professionals and without restriction of access. In the PHP outpatient clinic, patients are referred for medical treatment according to specific criteria and active recruitment by consulting the laboratory database. Admission to the PHP outpatient clinic was based on serum creatinine greater than 1.8 mg/dL or eGFR < 30 mL/min/1.73 m^2^. Patients with acute kidney injury (AKI) and those on kidney replacement therapy (KRT) were not recruited.

The inclusion criteria for this study were patients ≥ 18 years of age who had ≥ 3 medical appointments and came to follow-up visits for ≥ 6 months. Patients who were discharged from the outpatient clinic, who received palliative care, with AKI, or whose medical records had incomplete information were excluded. Consultations that occurred between 2014 and 2019 were evaluated, and the maximum follow-up time was 5 years. The study was carried out according to the Declaration of Helsinki and was approved by the research ethics committees of the participating centers under certificate number 39377020.1.1001.5505.

### 2.2. Variables and Definitions

The variables of interest to this study were collected by the same team of researchers in both participating services. Demographic data (age, sex, and ethnicity) and information on the etiology of CKD (AH, DM, glomerulopathies, polycystic kidneys, and other/indeterminate) and the presence or absence of the following comorbidities were collected: AH (defined by a diagnosis in the medical record or use of antihypertensive drugs), DM (defined by a diagnosis in the medical record or use of oral antidiabetic drugs or insulin), dyslipidemia (defined by a diagnosis in the medical record or use of statins or fibrates), coronary artery disease (history of angina or myocardial infarction, coronary stent implantation, and myocardial revascularization), cerebrovascular disease (history of stroke or transient ischemic attack), urolithiasis, chronic atrial fibrillation, and chronic obstructive pulmonary disease (defined by diagnosis in the medical record). Patients were considered to have AKI (and were therefore excluded from the study) when the serum creatinine value at the first visit fell by 0.3 mg/dL or more at subsequent visits and when the eGFR at subsequent visits remained > 60 mL/min/1.73 m^2^ (absence of CKD). At the beginning of the follow-up (baseline), the following data were obtained: blood pressure, number of antihypertensive drugs, serum creatinine concentration, and proteinuria. Proteinuria was categorically defined as the presence of one or more of the following: urine content with protein 1+ or more, albumin/creatinine ratio (ACR) > 30 mg/g, albuminuria > 30 mg/24 h, and proteinuria > 150 mg/24 h. This variable was classified as follows: absent or mild proteinuria (undetectable protein or traces by dipstick in isolated urine sample, ACR < 30 mg/g, albuminuria < 30 mg/24 h, and proteinuria < 150 mg/24 h), moderate proteinuria (protein 1+, ACR 30–300 mg/g, albuminuria 30–300 mg/24 h, and proteinuria 150–500 mg/24 h), and severe proteinuria (protein 2+ or 3+, ACR > 300 mg/g, albuminuria > 300 mg/24 h, and proteinuria > 500 mg/24 h) [18].

The use or nonuse of the following medications during follow-up was recorded: angiotensin-converting enzyme inhibitors (ACEIs) or angiotensin receptor blockers (ARBs), sodium-glucose-linked transporter-2 (SGLT-2) inhibitors, statins, acetylsalicylic acid, epoetin alfa, calcitriol, cholecalciferol, calcium carbonate, sevelamer, sodium bicarbonate, spironolactone, and beta-blockers.

### 2.3. Study Outcomes

The quality indicators evaluated were the percentages of patients with AH but blood pressure <140 × 90 mmHg, DM but HbA1c < 7.5%, and dyslipidemia but LDL-C < 100 mg/dL. In addition to these indicators, the percentages of patients with hemoglobin < 10 g/dL, serum potassium < 5.5 mEq/L, phosphorus < 4.5 mg/dL, 25-hydroxyvitamin D > 30 ng/mL, and controlled parathyroid hormone (PTH) were calculated. Furthermore, the percentage of patients who started hemodialysis with functioning AVF was evaluated [19].

AH was considered controlled when the mean of the last three systolic and diastolic blood pressure recordings was lower than 140 mmHg and 90 mmHg, respectively. Control of HbA1c, LDL-C, hemoglobin, serum potassium, phosphorus, and 25-hydroxyvitamin D was evaluated by the simple mean of the results over time. Blood pressure and HbA1c control were evaluated only in patients with AH and DM, respectively. As previously described, PTH was considered controlled when it decreased, remained steady, or increased < 30% compared to baseline [20].

The eGFR was calculated using the 2009 CKD Epidemiology Collaboration equation (CKD-EPI) and is expressed in mL/min/1.73 m^2^ body surface area [21]. The choice to use the CKD-EPI 2009 equation (without adjusting for ethnicity), and not the new 2021 equation, was due to previous studies validating this formula for the Brazilian population [22, 23]. The progression of CKD was calculated by dividing the difference between the first and last eGFR results by the follow-up time (expressed as mL/min/year). Thus, negative values indicated improvement in kidney function, and positive values corresponded to worsening of kidney function. A CKD progression rate less than −1.1 mL/min/year was classified as regression, a CKD progression rate between −1.0 and 1.0 mL/min/year was considered stable kidney function, a CKD progression rate between 1.1 and 4.0 mL/min/year was considered slow progression, and a value > 4.0 mL/min/year was considered rapid progression [24].

### 2.4. Statistical Analysis

Categorical variables are described as raw numbers and percentages. Quantitative variables with a normal distribution (according to the Kolmogorov–Smirnov test) are expressed as mean ± standard deviation and those with a nonnormal distribution are expressed as median (interquartile range). The frequency comparison was performed using the *χ*^2^ or Fisher test, as appropriate. Intergroup comparisons for quantitative variables were performed using Student's *t*-test or the Mann‒Whitney *U* test for normally and nonnormally distributed data, respectively.

Linear mixed-effects models were used to evaluate the evolution over time of eGFR between groups. The multivariate model was adjusted for baseline age, ethnicity, proteinuria, primary diagnosis of glomerulopathies, AH, DM, dyslipidemia, and systolic and diastolic blood pressure levels. Statistical analysis was performed with SPSS 20.0 (IBM Corp., Armonk, NY, USA) and STATA 17 (STATA Corp., College Station, TX, USA). A *p* value < 0.05 was considered significant.

## 3. Results and Discussion

### 3.1. Results

**Figure 1:**
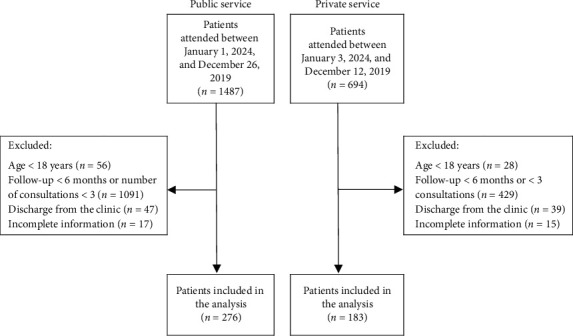
Flowchart of patient inclusion.

Between 2014 and 2019, 1487 patients were treated at the UHS nephrology service and 694 at the PHP. After excluding patients younger than 18 years (*n* = 84), with a follow-up time of less than 6 months (*n* = 1520), who were discharged from the outpatient clinic (*n* = 86), and with incomplete information (*n* = 32), the patients included in the final analysis were 276 from the UHS service and 183 from PHP (Figure 1).

Compared to PHP patients, UHS patients were older (63.4 [50.7–72.4] vs. 59.7 [48.3–71.3] years, *p* = 0.041) and presented as follows: a lower proportion of white individuals (32.6% vs. 51.9%), a higher proportion of mixed race individuals (26.8% vs. 4.9%), a higher prevalence of DM-caused CKD (44.2% vs. 29.8%), a lower prevalence of primary diagnosis of glomerulopathies (4.3% vs. 20.8%), a higher number of comorbidities (2 [2, 3] vs. 1 [1–3], *p* < 0.001), and a higher prevalence of AH (91.7% vs. 84.7%, *p* = 0.020) and dyslipidemia (58.3% vs. 38.3%, *p* < 0.001). At baseline, UHS patients had higher diastolic blood pressure (83 ± 13 vs. 81 ± 14 mmHg, *p* = 0.044), lower serum creatinine (1.60 [1.20–2.10] vs. 2.10 [1.60–2.80] mg/dL, *p* < 0.001), greater serum hemoglobin (12.8 [11.5–14.0] vs. 12.1 [10.6–13.4] g/dL, *p* = 0.003), and higher glycated hemoglobin level (7.2 [6.1–8.8] vs. 6.2 [5.6–7.3] %, *p* < 0.001) than PHP patients. Among patients with proteinuria, a lower proportion of patients in UHS had severe proteinuria than patients in PHP (36.6% vs. 49.2%, respectively, *p* < 0.001). There were no significant differences between the groups in the sex distribution, the prevalence of other comorbidities, presence of proteinuria (categorized as present or not), or the results of the following measured at baseline: LDL-C, potassium, 25-hydroxyvitamin D, phosphorus, and PTH (Table 1).

During the follow-up period, compared to PHP patients, UHS patients used more ACEIs or ARBs (74.6% vs. 52.5%, *p* < 0.001) and acetylsalicylic acid (41.3% vs. 30.1%, *p*=0.014) and used fewer SGLT-2 inhibitors (0.4% vs. 13.7%, *p* < 0.001), epoetin alfa (9.8% vs. 29.9%, *p* < 0.001), calcium carbonate (1.8% vs. 16.4%, *p* < 0.001), sodium bicarbonate (4.7% vs. 13.1%, *p*=0.001), spironolactone (5.4% vs. 16.4%, *p* < 0.001), and beta-blockers (35.5% vs. 57.9%, *p* < 0.001). CKD progression was faster in patients with UHS than in patients with PHP (2.3 [−0.1; 5.9] vs. 1.1 [−1.9; 4.6] mL/min/year, *p*=0.005). There were no significant differences between the groups in the use of statins, calcitriol, cholecalciferol, or sevelamer. The following measures also did not differ between the groups: follow-up time (3.6 [2.3–5.1] years in UHS vs. 3.2 [1.6–5.2] in PHP, *p*=0.413), the percentage of patients referred for outpatient RRT (17.0% in UHS vs. 19.1% in PHP, *p*=0.566), and the RRT method (hemodialysis in 93.6% of patients in UHS vs. 100.0% in PHP, *p*=0.129) (Table 2).

Compared to PHP patients, UHS had a lower percentage of patients with DM and HbA1c < 7.5% (46.1% vs. 61.2%, *p*=0.031), a lower percentage of people with serum potassium < 5.5 mEq/L (90.4% vs. 95.6%, *p*=0.040), and a lower initiation of hemodialysis with a functioning AVF (9.1% vs. 54.3%, *p* < 0.001). There were no significant difference between the groups in AH control (59.7% in UHS vs. 66.7% in PHP, *p*=0.171), dyslipidemia control (38.3% vs. 49.3%, *p*=0.125), or PTH control (85.6% vs. 84.8%, *p*=833), nor in the percentage of patients with hemoglobin < 10 g/dL (95.2% in UHS vs. 93.4% in PHP, *p*=0.424), phosphorus < 4.5 mg/dL (78.5% vs. 72.2%, *p*=0.158), or 25-hydroxyvitamin D > 30 ng/mL (28.4% vs. 36.5%, *p*=0.111) (Table 3).

**Figure 2:**
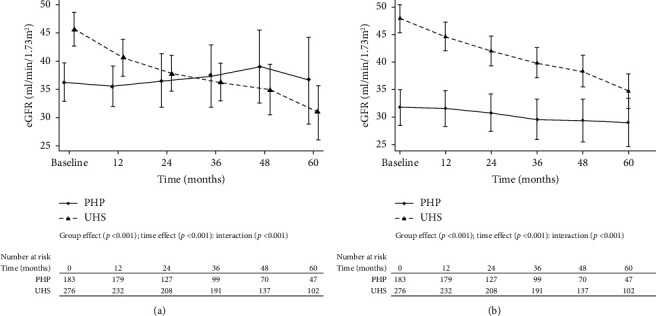
Chronic kidney disease progression in UHS and PHP over time. (a) Unadjusted model. (b) Adjusted for age, ethnicity, proteinuria, primary diagnosis of glomerulopathies, arterial hypertension, diabetes, dyslipidemia, and baseline systolic and diastolic blood pressure. eGFR, estimated glomerular filtration rate; PHP, private health plan; UHS, Unified Health System. The number of eGFR measurements is equal to the number of patients at risk in each time interval.

Using a linear mixed-effects model, a significant difference was observed in the longitudinal change of eGFR between UHS and PHP (group effect, *p* < 0.001; time effect, *p* < 0.001; interaction, *p* < 0.001; Figure 2(a)). The UHS exhibited a faster decline in eGFR than the PHP group. Similar results were obtained after adjustment for age, ethnicity, baseline proteinuria, primary diagnosis of glomerulopathy, AH, DM, dyslipidemia, and baseline systolic and diastolic blood pressure levels (group effect, *p* < 0.001; time effect, *p* < 0.001; interaction, *p* < 0.001; Figure 2(b)).

### 3.2. Discussion

The present study showed that patients with nondialysis CKD in the UHS were older, had more comorbidities, and had a faster disease progression than those in the PHP, although the former had a higher eGFR at the beginning of follow-up. UHS patients used more ACEIs or ARBs than PHP patients with PHP, but had lower use of SGLT-2 inhibitors, epoetin alfa, and calcium carbonate. The performance in the care quality indicators was, in general, worse in the UHS than in the PHP.

The UHS is one of the largest public health policies ever implemented worldwide. More than 200 million Brazilian citizens have access to it regardless of age, comorbidities, income, or employment status [25]. On the other hand, the actions and services of supplementary (or private) health require the contracting of a PHP on an individual, family, or company basis [26]. Thus, in general, individuals with PHP in Brazil are economically active and have better social and health conditions than patients with exclusive coverage from the UHS. This could be an explanation for the difference in age and comorbidities between UHS and PHP patients found in our study. A previous Brazilian study conducted in a population at risk of CKD also revealed a higher mean age and a higher burden of disease in patients treated in UHS than those treated in PHP [27].

The difference in baseline eGFR between UHS patients and PHP patients may be attributed to the recruitment methods employed for the evaluated secondary care services. PHP evaluated has a laboratory-based CKD surveillance system that actively identifies individuals with eGFR < 30 mL/min/1.73 m^2^ for enrollment in the multidisciplinary care program [28]. In contrast, UHS referrals are made exclusively based on the individual judgment of primary care physicians [29]. At least 10% of patients referred to nephrologists could potentially be managed through primary health care, indicating suboptimal use of specialist consultations and deficiency in proper matrix support and counter-referral by nephrologists [27, 30, 31].

The increased utilization of ACEIs or ARBs in UHS (75%) compared to PHP (53%) may be attributed to the availability of these drugs in the public network at no cost [32]. Their low use in patients with PHP indicates the need for professional training, as these are known to be nephroprotective medications [33]. According to an international multicenter study (CKD Outcomes and Practice Patterns Study, CKDopps), 80% of CKD patients treated through multidisciplinary services in Germany, 77% in France, 66% in Brazil, and 52% in the United States use renin–angiotensin system inhibitors [34].

The increased use of SGLT-2 inhibitors in supplementary health care, although in a small percentage of patients, may be attributed to the better socioeconomic conditions of PHP patients compared to UHS patients [35]. Indeed, these medications were not available in the UHS at the time of the study. Reduced use of beta-blockers and spironolactone in patients with UHS compared to PHP patients may be associated with better evaluation and treatment of cardiovascular disease in the latter group [36, 37]. Although UHS individuals are older and have more comorbidities, our study did not reveal a difference in the prevalence of coronary artery disease or heart failure between the two groups of patients.

A possible explanation for the increased utilization of epoetin alfa, calcium carbonate, and sodium bicarbonate in PHP patients compared to UHS patients could be the poorer kidney function observed at the beginning of follow-up in PHP patients. In fact, patients with UHS had a higher baseline mean level of hemoglobin compared to patients with PHP. However, it should be noted that all of these medications are freely available in the Brazilian public health network [32].

The control of AH observed in this study (62%) was better than that reported in a previous study conducted in a public tertiary hospital (51%), but it was less than that reported in clinics participating in Brazilian CKDopps (76%) [34, 38, 39]. The proportions of patients with DM and serum potassium control and the referral rate for hemodialysis with a functioning AVF were higher in PHP than in UHS. The lack of universal access to AVF creation in UHS for patients receiving conservative CKD treatment poses a significant barrier to the implementation of this line of care in individuals with advanced CKD [35]. Despite better baseline kidney function, patients with UHS showed a faster decline in eGFR over time than patients with PHP. This difference persisted even after adjustment for known progression risk factors of CKD, such as age, ethnicity, proteinuria, AH, DM, and dyslipidemia [18, 22]. Together, these results could be attributed to the involvement of the multidisciplinary team in the care of chronic and complex patients, particularly in the evaluated PHP, and the clinics participating in CKDopps [39–41]. Actually, a systematic review with meta-analysis showed that the outcomes of patients with advanced CKD can be improved when care is multidisciplinary, but not when it is focused solely on nephrologists [42].

The strengths of this study are the 5-year follow-up, the use of real-world data, and its relevance for improving public policies to address CKD. The limitations of this research were its retrospective design, the lack of data on indications and contraindications for the use of the evaluated drugs, the absence of data on adherence to appointments and tests requested, and the unavailability of information on the laboratory equipment and methods used by the participating institutions. However, it is unlikely that the type of equipment caused the baseline laboratory differences or interfered with the results of the longitudinal variation in eGFR between groups as all measurements were automated and standardized. The laboratory differences between groups are consistent with the differences in age and comorbidities of the patients. Despite the role of ACEIs/ARBs in slowing the progression of CKD, they were not included in our multivariate longitudinal eGFR analysis due to the lack of information on adherence to these medications. Furthermore, the presence of residual or unmeasured differences in socioeconomic, educational status, demographic, and comorbidity profiles at the beginning of the study prevents the complete elimination of bias in the comparison between the outcomes of patients with UHS and PHP.

In summary, improving the quality of care for patients with CKD is essential in both evaluated services through the implementation of multidisciplinary care, as well as other treatment resources, for example, additional types of nephroprotective drugs. This approach could have a positive impact on the management of comorbidities and facilitate the preparation for the initiation of hemodialysis. In the UHS, on which approximately 75% of the Brazilian population is exclusively dependent, it is imperative to overcome barriers in the care of patients with advanced CKD. These challenges include, among others, a laboratory-based surveillance system, universal access to multidisciplinary care, monitoring of quality indicators, and improved access to the creation of AVF.

## Figures and Tables

**Table 1:** The baseline characteristics of the patients included.

Variable	All (*n* = 459)	UHS (*n* = 276)	PHP (*n* = 183)	*p* value∗
Age (years)	61.5 (49.8–72.3)	63.4 (50.7–72.4)	59.7 (48.3–71.3)	0.04
Male sex, % (*n*)	52.3 (240)	50.0 (138)	55.7 (102)	0.25
Ethnicity				
White, % (*n*)	40.3 (185)	32.6 (90)	51.9 (95)	< 0.01
Brown, % (*n*)	18.1 (83)	26.8 (74)	4.9 (9)	
Black, % (*n*)	4.8 (22)	3.3 (9)	7.1 (13)	
Asian, % (*n*)	0.7 (3)	0.7 (2)	0.5 (1)	
Unknown, % (*n*)	36.2 (166)	36.6 (101)	35.5 (65)	
Etiology of chronic kidney disease				
Arterial hypertension, % (*n*)	38.8 (178)	37.7 (104)	40.4 (74)	< 0.01
Diabetes, % (*n*)	38.3 (176)	44.2 (122)	29.5 (54)	
Glomerulopathy, % (*n*)	10.9 (50)	4.3 (12)	20.8 (38)	
Polycystic kidneys, % (*n*)	3.9 (18)	4.7 (13)	2.7 (5)	
Undetermined/others, % (*n*)	8.1 (37)	10.0 (25)	6.6 (12)	
Number of comorbidities	2 (2-3)	2 (2-3)	1 (1–3)	< 0.01
Arterial hypertension, % (*n*)	88.9 (408)	91.7 (253)	84.7 (155)	0.02
Diabetes, % (*n*)	51.6 (237)	55.1 (152)	46.4 (85)	0.07
Dyslipidemia, % (*n*)	50.3 (231)	58.3 (161)	38.3 (70)	< 0.01
Urinary lithiasis, % (*n*)	15.7 (72)	18.1 (50)	12.0 (22)	0.08
Heart failure, % (*n*)	15.7 (72)	17.4 (48)	13.1 (24)	0.22
Coronary artery disease, % (*n*)	13.1 (60)	13.0 (36)	13.1 (24)	0.98
Cerebrovascular disease, % (*n*)	10.5 (48)	12.7 (35)	7.1 (13)	0.06
Chronic atrial fibrillation, % (*n*)	4.8 (22)	3.6 (10)	6.6 (12)	0.15
Chronic lung disease, % (*n*)	5.0 (23)	6.2 (17)	3.3 (6)	0.17
Systolic blood pressure (mmHg)	135 ± 23	134 ± 23	137 ± 25	0.28
Diastolic blood pressure (mmHg)	82 ± 13	83 ± 13	81 ± 14	0.04
Number of antihypertensive drugs	2 (1–3)	2 (1–3)	2 (1–3)	0.98
Laboratory tests at *baseline*				
Creatinine (mg/dL)	1.75 (1.33–2.30)	1.60 (1.20–2.10)	2.10 (1.60–2.80)	< 0.01
eGFR (ml/min/1.73 m^2^)	34.8 (24.0–50.9)	39.1 (28.6–54.8)	29.9 (21.5–42.0)	< 0.01
≥ 90, % (*n*)	6.3 (29)	7.2 (20)	4.9 (9)	< 0.01
60–89, % (*n*)	10.7 (49)	12.3 (34)	8.2 (15)	
45–59, % (*n*)	15.9 (73)	20.7 (57)	8.7 (16)	
30–44, % (*n*)	29.0 (133)	30.1 (83)	27.3 (50)	
15–29, % (*n*)	32.5 (149)	27.5 (76)	39.9 (73)	
< 15, % (*n*)	5.7 (26)	2.2 (6)	10.9 (20)	
Hemoglobin (g/dL)	12.3 (11.2–13.5)	12.8 (11.5–14.0)	12.1 (10.6–13.4)	< 0.01
LDL-cholesterol (mg/dL)	120 ± 60	115 ± 46	125 ± 70	0.19
Glycated hemoglobin (%)	6.6 (5.8–8.0)	7.2 (6.1–8.8)	6.2 (5.6–7.3)	< 0.01
Potassium (mEq/L)	4.7 (4.3–5.1)	4.7 (4.3–5.1)	4.7 (4.2–5.1)	0.73
25-hydroxyvitamin D (ng/mL)	25 ± 11	25 ± 9	25 ± 12	0.64
Phosphorus (mg/dL)	4.0 ± 0.8	3.9 ± 0.8	4.1 ± 0.8	0.06
Parathyroid hormone (pg/mL)	74 (43–129)	90 (47–147)	62 (40–109)	0.09
Proteinuria, % (*n*)	63.6 (292)	64.5 (178)	62.3 (114)	0.63
Mild or absent, % (*n*)	36.4 (167)	35.5 (98)	37.7 (69)	< 0.01
Moderate, % (*n*)	22.0 (101)	27.9 (77)	13.1 (24)	
Severe, % (*n*)	41.6 (191)	36.6 (101)	49.2 (90)	

*Note:* Data are presented as median (interquartile ranges), mean (standard deviation), or percentages.

Abbreviations: eGFR, estimated glomerular filtration rate; LDL, low-density lipoprotein; PHP, private health plan; UHS, Unified Health System.

^∗^UHS vs. PHP.

**Table 2:** Use of medications and kidney function during follow-up.

Variable	All (*n* = 459)	UHS (*n* = 276)	PHP (*n* = 183)	*p* value1
Medications used during follow-up2				
ACEIs or ARBs, % (*n*)	65.8 (302)	74.6 (206)	52.5 (96)	< 0.01
SGLT-2 inhibitor, % (*n*)	5.7 (26)	0.4 (1)	13.7 (25)	< 0.01
Statin, % (*n*)	61.2 (281)	63.0 (174)	58.5 (107)	0.33
Acetylsalicylic acid, % (*n*)	36.8 (169)	41.3 (114)	30.1 (55)	0.01
Epoetin alfa, % (*n*)	17.4 (80)	9.8 (27)	29.9 (53)	< 0.01
Calcitriol, % (*n*)	7.2 (33)	5.8 (16)	9.3 (17)	0.16
Cholecalciferol, % (*n*)	28.5 (131)	29.7 (82)	26.8 (49)	0.50
Calcium carbonate, % (*n*)	7.6 (35)	1.8 (5)	16.4 (30)	< 0.01
Sevelamer, % (*n*)	3.3 (15)	2.2 (6)	4.9 (9)	0.11
Sodium bicarbonate, % (*n*)	8.1 (37)	4.7 (13)	13.1 (24)	< 0.01
Spironolactone, % (*n*)	9.8 (45)	5.4 (15)	16.4 (30)	< 0.01
Beta-blocker, % (*n*)	44.4 (204)	35.5 (98)	57.9 (106)	< 0.01
Follow-up time (years)	3.4 (2.0–5.1)	3.6 (2.3–5.1)	3.2 (1.6–5.2)	0.43
CKD progression (mL/min/year)^3^	1.8 (−0.9; 5.5)	2.3 (-0.1;5.9)	1.1 (-1.9; 4.6)	< 0.01
Stable kidney function or regression, % (*n*)^a^	42.3 (194)	37.3 (103)	49.7 (91)	0.02
Slow progression, % (*n*)^b^	25.3 (116)	26.4 (73)	23.5 (43)	
Rapid progression, % (*n*)^c^	32.5 (149)	36.2 (100)	26.8 (49)	
Referral to outpatient KRT, % (*n*)	17.9 (82)	17.0 (47)	19.1 (35)	0.57
Hemodialysis, % (*n*)	96.3 (79)	93.6 (44)	100.0 (35)	0.13
Peritoneal dialysis, % (*n*)	3.7 (3)	6.4 (3)	0.0 (0)	
Pre-emptive kidney transplantation, % (*n*)	0.0 (0)	0.0 (0)	0.0 (0)	

*Note:* Data are presented as median (interquartile range) or percentage.

Abbreviations: ACEIs, angiotensin-converting enzyme inhibitors; ARBs, angiotensin receptor blockers; CKD, chronic kidney disease; KRT, kidney replacement therapy; PHP, private health plan; SGLT-2, sodium-glucose-linked transporter-2; UHS, Unified Health System.

^1^UHS vs. PHP.

^2^The use of medications was considered present if they were documented in the charts during any follow-up visit.

^3^Calculated with estimated glomerular filtration rate (eGFR) measures in the first and last patient visit.

^a^Elevation, maintenance, or < 1.0 mL/min/year reduction in the estimated glomerular filtration rate (eGFR).

^b^Reduction in eGFR between 1.1 and 4.0 mL/min/year.

^c^Reduction in eGFR > 4.0 mL/min/year.

**Table 3:** Quality indicators of secondary health care in chronic kidney disease.

Indicator	All (*n* = 459)			UHS (*n* = 276)			PHP (*n* = 183)			*p* value∗
	Numerator (*n*)	Denominator (*n*)	Result (%)	Numerator (*n*)	Denominator (*n*)	Result (%)	Numerator (*n*)	Denominator (*n*)	Result (%)	
Patients with AH and BP < 140 × 90 mmHga	254	408	62.3	151	253	59.7	103	155	66.7	0.17
Patients with DM and HbA1c < 7.5%^b^	111	213	52.1	59	128	46.1	52	85	61.2	0.03
Patients with DLP and LDL-C < 100 mg/dLb	93	223	41.7	59	154	38.3	34	69	49.3	0.13
Patients with Hb < 10 g/dLb	410	434	94.5	239	251	95.2	171	183	93.4	0.42
Patients with K < 5.5 mEq/lb	401	433	92.6	226	250	90.4	175	183	95.6	0.04
Patients with P < 4.5 mg/dLb	269	356	75.6	142	181	78.5	127	175	72.0	0.16
Patients with 25-hydroxyvit. D > 30 ng/mLb	110	342	32.2	52	183	28.4	58	159	36.5	0.11
Patients with controlled PTHc	283	332	85.2	149	174	85.6	134	158	84.8	0.83
Patients who initiated HD with AVF	23	79	29.1	4	44	9.1	19	35	54.3	< 0.01

Abbreviations: 25-hydroxyvit. D, 25-hydroxyvitamin D; AH, arterial hypertension; AVF, arteriovenous fistula; BP, blood pressure; DM, diabetes mellitus; DLP, dyslipidemia; HbA1c, glycosylated hemoglobin; HD, hemodialysis; Hb, hemoglobin; LDL-C, low-density lipoprotein cholesterol; PTH, parathyroid hormone; PHP, private health plan; P, serum phosphorus; K, serum potassium; UHS, Unified Health System.

^∗^UHS vs. PHP.

^a^Mean of the measurements of the last 3 consultations.

^b^Mean of the results of exams performed during follow-up.

^c^Reduction, stability, or < 30% elevation compared to baseline.

## Data Availability

The datasets used and/or analyzed during this study are available from the corresponding author upon reasonable request and after adherence to local research ethics regulations.
